# Retracted: Drop Height Impacts the Lower Limb Elastic Energy's Utilization for Male High Jumpers: A Experimental Research from Biomechanics

**DOI:** 10.1155/2023/9839748

**Published:** 2023-02-05

**Authors:** Applied Bionics and Biomechanics

*Applied Bionics and Biomechanics* has retracted the article titled “Drop Height Impacts the Lower Limb Elastic Energy's Utilization for Male High Jumpers: A Experimental Research from Biomechanics” [1] due to concerns that the peer review process has been compromised.

Following an investigation conducted by the Hindawi Research Integrity team [2], significant concerns were identified with the peer reviewers assigned to this article; the investigation has concluded that the peer review process was compromised. We therefore can no longer trust the peer review process and the article is being retracted with the agreement of the Chief Editor.

The authors do not agree to the retraction.
